# First Complete Genome of the Thermophilic Polyhydroxyalkanoates-Producing Bacterium *Schlegelella thermodepolymerans* DSM 15344

**DOI:** 10.1093/gbe/evab007

**Published:** 2021-01-12

**Authors:** Jana Musilova, Xenie Kourilova, Matej Bezdicek, Martina Lengerova, Stanislav Obruca, Helena Skutkova, Karel Sedlar

**Affiliations:** 1 Department of Biomedical Engineering, Faculty of Electrical Engineering and Communication, Brno University of Technology, Czech Republic; 2 Department of Food Chemistry and Biotechnology, Faculty of Chemistry, Brno University of Technology, Czech Republic; 3 Department of Internal Medicine—Hematology and Oncology, University Hospital Brno, Czech Republic

**Keywords:** de novo assembly, hybrid assembly, functional annotation, PHA

## Abstract

*Schlegelella thermodepolymerans* is a moderately thermophilic bacterium capable of producing polyhydroxyalkanoates—biodegradable polymers representing an alternative to conventional plastics. Here, we present the first complete genome of the type strain *S. thermodepolymerans* DSM 15344 that was assembled by hybrid approach using both long (Oxford Nanopore) and short (Illumina) reads. The genome consists of a single 3,858,501-bp-long circular chromosome with GC content of 70.3%. Genome annotation identified 3,650 genes in total, whereas 3,598 open reading frames belonged to protein-coding genes. Functional annotation of the genome and division of genes into clusters of orthologous groups revealed a relatively high number of 1,013 genes with unknown function or unknown clusters of orthologous groups, which reflects the fact that only a little is known about thermophilic polyhydroxyalkanoates-producing bacteria on a genome level. On the other hand, 270 genes involved in energy conversion and production were detected. This group covers genes involved in catabolic processes, which suggests capability of *S. thermodepolymerans* DSM 15344 to utilize and biotechnologically convert various substrates such as lignocellulose-based saccharides, glycerol, or lipids. Based on the knowledge of its genome, it can be stated that *S. thermodepolymerans* DSM 15344 is a very interesting, metabolically versatile bacterium with great biotechnological potential.

**Significance**

The type strain *Schlegelella thermodepolymerans* DSM 15344 is a thermophilic bacterium capable of production of polyhydroxyalkanoates (PHA)—microbial polyesters representing “green” alternative to petrochemical polymers. The fact that the bacterium grows and thrives at elevated temperatures brings numerous benefits (e.g., reduced risk of contamination) which might positively influence the process of PHA production. Moreover, due to enormous metabolic flexibility, *S. thermodepolymerans* DSM 15344 is capable of PHA production from human food chain noncompeting substrates such as lignocelluloses and other waste streams of the agro-food industry. Even though the strain DSM 15344 is biotechnologically promising, genomic information on this strain is scarce. Here, we describe the first complete genome of the *S. thermodepolymerans* DSM 15344 that will facilitate studies on its PHA metabolism and enable us to further investigate and improve its biotechnological potential.

## Introduction

Polyhydroxyalkanoates (PHA) are polyesters of hydroxyalkanoic acids. As the PHA are produced naturally by microbial fermentation, they can be regarded as an environmental friendly alternative to petroleum-based polymers (Muhammadi et al. 2015; Sabapathy et al. 2020). Although some facts regarding PHA fermentation are known, for example, microorganisms use PHA to store unused energy and carbon into cytoplasm in a form of intracellular granules and these granules help the organism to cope with stressors (Obruca et al. 2018), additional basic knowledge is needed to establish viable industrial processes. Although production of bioplastics is considered to be the future way and inseparable part of circular economy, less than 1% of the total plastic production comes from bioplastics industry (Shogren et al. 2019).

The type strain *S. thermodepolymerans* DSM 15344 is a thermophilic, Gram-negative bacterium that was originally investigated for its ability to degrade extracellular PHA materials such as copolymers of 3-hydroxybutyrate and 3-mercaptopropionate (Elbanna et al. 2003). So far, two draft genome assemblies of the strain were published. The assembly available under the GenBank accession number GCA_002933415.1 submitted by Zhejiang Academy of Agricultural Sciences consists of 48 contigs with N50 length of 174,537 bp and the assembly GCA_003349825.1 by DOE Joint Genome Institute contains 28 scaffolds with N50 length of 324,832 bp. Although these represent relatively high-quality draft assemblies, probably due to missing high-quality complete genome assembly and functional annotation of the genome, other important features of the strain remained hidden. Only recently, its ability to produce PHA was reported together with the unique capability of xylose utilization (Kourilova et al. 2020). Optimal growth temperature of *S. thermodepolymerans* DSM 15344, 55 °C, reduces the risk of microbial contamination; therefore, the strain presents an ideal organism for utilization in the “Next Generation Industrial Biotechnology” concept in which biotechnological process is conducted under unsterile conditions (Chen and Jiang 2018). In this article, we present its first high-quality complete genome sequence, which is currently a reference sequence for *S. thermodepolymerans* species in GenBank database. We annotated the genome, predicted the operon structure, and searched for prophage DNA and CRISPR arrays.

## Materials and Methods

### Growth Conditions, DNA Extraction, and Sequencing

*Schlegelella thermodepolymerans* DSM 15344 was obtained from Leibniz Institute DSMZ-German Collection of Microorganisms and Cell Cultures Braunschweig, Germany. Optimal temperature for bacterial growth was 55 °C. The inoculum was developed in nutrient-rich medium Nutrient Broth (HiMedia, India) containing peptone 10.0 g/l, beef extract 10.0 g/l, and sodium chloride 5.0 g/l. After 24 h, 5% of the bacterial suspension was inoculated into a mineral salt medium composed of Na_2_HPO_4_ · 12 H_2_O (9.0 g/l), KH_2_PO_4_ (1.5 g/l), NH_4_Cl (1.0 g/l), MgSO_4_ · 7 H_2_O (0.2 g/l), CaCl_2_ · 2 H_2_O (0.02 g/l), Fe^(III)^NH_4_citrate (0.0012 g/l), yeast extract (0.5 g/l), 1 ml/l of microelements solution (EDTA [50.0 g/l], FeCl_3_ · 6 H_2_O [13.8 g/l], ZnCl_2_ [0.84 g/l], CuCl_2_ · 2 H_2_O [0.13 g/l], CoCl_2_ · 6 H_2_O [0.1 g/l], MnCl_2_ · 6 H_2_O [0.016 g/l], H_3_BO_3_ [0.1 g/l], dissolved in distilled water), and a xylose (20.0 g/l) as a carbon substrate. *Schlegelella thermodepolymerans* DSM 15344 was cultivated in mineral salt medium under the same conditions as the inoculum.

Genomic DNA was extracted using MagAttract HMW DNA Kit (Qiagene, NL). The DNA purity was checked using NanoDrop (Thermo Fisher Scientific), the concentration was measured using Qubit 2.0 Fluorometr (Thermo Fisher Scientific), and the proper length of the isolated DNA was confirmed using Agilent 4200 TapeStation (Agilent technologies). The sequencing library for Oxford Nanopore sequencing was prepared using Ligation sequencing 1D Kit (Oxford Nanopore Technologies, UK). The sequencing was performed using the R9.4.1 flowcell and the MinION platform (Oxford Nanopore Technologies). The sequencing library for short-read sequencing was prepared using KAPA HyperPlus kit and was carried out using Miseq Reagent Kit v2 (500 cycles) and Illumina MiSeq platform (Illumina).

### Genome Assembly

The Nanopore reads were basecalled using Guppy v3.4.4 (https://community.nanoporetech.com, last accessed September 18, 2020), and quality was checked using MinIONQC (Lanfear et al. 2019). Subsequently, the reads were assembled with Flye v2.8.1 (https://github.com/fenderglass/Flye, last accessed October 2, 2020). Polishing was done using Racon v1.4.13 (Vaser et al. 2017) and Medaka (https://github.com/nanoporetech/medaka, last accessed October 8, 2020); auxiliary PAF files were generated using minimap2 (Li 2018). The Illumina paired-end (PE) reads were initially quality-checked using FastQC v0.11.5 and MultiQC v1.7 (Ewels et al. 2016). Trimmomatic v1.36 (Bolger et al. 2014) was subsequently used for the adapter and quality trimming. In the next step, trimmed Illumina PE reads were mapped to the Nanopore initial assembly using BWA v07.17 (Hi 2013). Finally, the obtained assembly was polished using Pilon v1.23 (Walker et al. 2014); auxiliary BAM files were obtained using SAMtools (Li et al. 2009). As the last step, the final assembly was rearranged according to the replication origin (oriC) identified using Ori-finder (Luo et al. 2019), so the *DnaA* gene is the first gene.

### Genome Annotation and Analysis

Genome annotation was done through the NCBI Prokaryotic Genome Annotation Pipeline (PGAP) (Tatusova et al. 2016). An operon prediction was performed using Operon-mapper (Taboada et al. 2018), and the results were manually inserted into the genome record. Functional annotation of the protein-coding genes was performed by classifying them into clusters of orthologous groups (COG) from the eggNOG database using the eggNOG-mapper (Huerta-Cepas et al. 2019). Chromosomal map of the circular genome was subsequently produced with the Artemis (Rutherford et al. 2000)-integrated DNAPlotter (Carver et al. 2009). Prophage DNA was searched using Prophage Hunter (Song et al. 2019) and PHASTER (Arndt et al. 2016). Finally, the annotated genome sequence was further analyzed for presence of CRISPR loci using CRISPRDetect tool (Biswas et al. 2016).

## Results and Discussion

### Genome Assembly and Properties

*Schlegelella thermodepolymerans* DSM 15344 initial genome assembly was reconstructed from nearly 1.8 million Oxford Nanopore Technologies reads with a median read length of 4.9 kb and finalized by mapping more than 2.4 million high-quality (average Phred score Q ≈ 35) Illumina read pairs (88% of all Illumina reads) to the initial assembly. Whole process resulted into the final assembly consisting of one circular chromosome with coverage exceeding 5,500×. The genome has been deposited at the DDBJ/EMBL/GenBank under accession number CP064338.1.

The genome length is 3,858,501 bp and contains 3,650 genes in total, divided into 1,729 operons. Most of the genes are protein-coding sequences (CDSs), but 33 pseudogenes were also found, which is less than 44 and 50 pseudogenes detected in previously published draft genome sequences PSNY00000000.1 and QQAP00000000.1, respectively. The GC content reached the value of 70.28% which is more than the average for Gram-negative bacteria (Li and Du 2014). However, it met our expectations, as it corresponded to the value 70.3% of the previously published draft genomes. High GC content can be associated with the adaptation of the bacterium to high-temperature environments. Although only single copies of rRNA genes were detected in draft genomes of *S. thermodepolymerans* DSM 15344, the complete genome sequence contains 5S, 16S, and 23S rRNA genes in duplicates. Moreover, copies of 16S and 23S rRNA genes differ in three and one positions, respectively. Such information is useful for future identification of *S. thermodepolymerans* in metagenomics studies and quantification of its abundance in microbial studies based on amplicon sequencing. The overall sequence features are summarized in table 1.

**Table 1:** Genomic Features of *Schlegelella themordepolymerans* DSM 15344

Feature	Chromosome
Length (bp)	3,858,501
GC content (%)	70.28
Genes	3,650
Operons	1,729
CDS	3,589
Pseudogenes	33
rRNA (5S, 16S, 23S)	2, 2, 2
tRNA	51
ncRNA	4

### Functional Annotation

The protein-coding genes were classified according to COG into 22 categories. In total, 2,576 CDSs were assigned a COG category with the most prevalent groups E—amino acid metabolism and transport containing 7.80% of the total number of CDS (280 out of 3,589) and C—energy production and conversion containing 7.52% of the total number of CDS (270 out of 3,589). This suggests that *S. thermodepolymerans* has a functional apparatus capable of utilizing a wide range of substrates as reported recently (Kourilova et al. 2020). Unfortunately, 9.33% (335 genes) were not assigned any COG and 18.89% (678 genes) were assigned group S with an unknown function. In fact, such a result was expected as only a little is known about genomes of thermophilic bacteria capable of PHA synthesis so far. (For details of each group, including the number of assigned genes assigned see supplementary table S1, Supplementary Material online.)

**Fig. 1:**
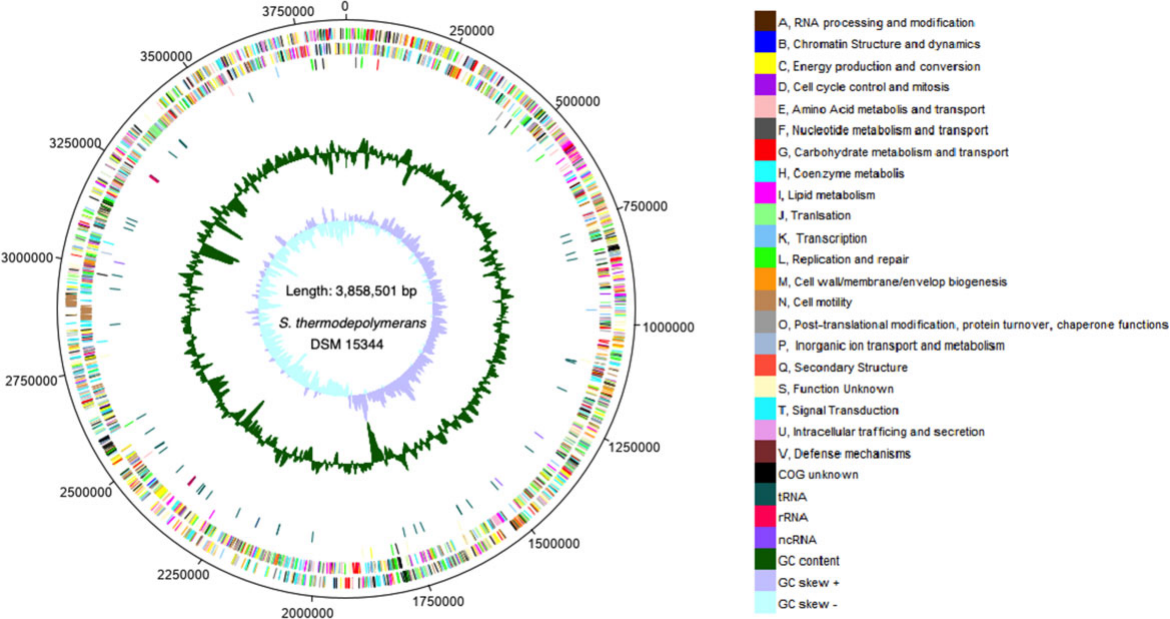
A chromosomal map of the *S. thermodepolymerans* DSM 15344 genome. The first two outermost circles represent CDSs on the forward and backward strands, respectively; the third circle represents pseudogenes. The colors represent the functional classification of COG. The fourth outermost circle represents RNA genes, distinguishing among tRNA, rRNA, and ncRNA. The inner area represents the GC content and GC skew.

The position of individual features in the circular genome is shown in figure 1. Each COG is marked with a different color. Moreover, RNAs are divided into tRNA, rRNA, and ncRNA categories and displayed in the fourth outermost circle.

Searching for viral DNA resulted only in inconclusively identified prophages. Although Prophage Hunter identified five putative prophages, PHASTER results consisted of a single incomplete prophage that overlapped with one candidate indentified by Prophage Hunter. None of these phages was identified as active. This is according to our expectations as phages are viruses for which temperature is a crucial factor for survivability (Nasser and Oman 1999). Optimal temperature for growth of the strain (55 °C) is too high for most phages (Farrell and Campbell 1969). Although a group of thermophilic phages also exists, they usually occur in specific environment (Jończyk et al. 2011) and were not identified in the *S. thermodepolymerans* DSM 15344 genome. Only a single 164-bp-long CRISPR array containing two spacer units was found in the *S. thermodepolymerans* DSM 15344 genome. Unfortunately, no *cas* or *cas*-like genes were found in its neighborhood. Nevertheless, this does not prevent the CRISPR-Cas9 being utilized for *S. thermodepolymerans* DSM 15344 genome editing as a foreign system could be used.

## Supplementary Material

Supplementary data are available at *Genome Biology and Evolution* online.

## Supplementary Material

evab007_Supplementary_Data
