# Supplementary material for: First Complete Genome of the Thermophilic Polyhydroxyalkanoates-Producing Bacterium Schlegelella thermodepolymerans DSM 15344
Source: Genome Biol Evol. 2021 Jan 12;13(6):evab007. doi: 10.1093/gbe/evab007 (PMC8023429; doi:10.1093/gbe/evab007)
Supplement: evab007_Supplementary_Data [file evab007_supplementary_data.pdf]

Suplementary table S1

Clusters of orthologous groups of *Schlegelella themordepolymerans* DSM 15344

COG class	Description	Gene count	Percentage
A	RNA processing and modification	1	0.03%
B	Chromatin Structure and dynamics	2	0.06%
C	Energy production and conversion	270	7.52%
D	Cell cycle control and mitosis	40	1.11%
E	Amino Acid metabolism and transport	280	7.80%
F	Nucleotide metabolism and transport	96	2.67%
G	Carbohydrate metabolism and transport	127	3.54%
H	Coenzyme metabolis	141	3.93%
I	Lipid metabolism	163	4.54%
J	Tranlsation	179	4.99%
K	Transcription	255	7.11%
L	Replication and repair	148	4.12%
M	Cell wall/membrane/envelop biogenesis	188	5.24%
N	Cell motility	95	2.65%
O	Post-translational modification, protein turnover, chaperone functions	120	3.34%
P	Inorganic ion transport and metabolism	165	4.60%
Q	Secondary Structure	75	2.09%
S	Function Unknown	678	18.89%
T	Signal Transduction	131	3.65%
U	Intracellular trafficing and secretion	70	1.95%
V	Defense mechanisms	30	0.84%
	COG unknown	335	9.33%
